# Medication-Related Osteonecrosis of the Jaw According to Antiresorptive Treatment Indication: Disease Severity and Associated Factors

**DOI:** 10.3390/jcm15218402

**Published:** 2026-10-28

**Authors:** Ivona Mihaela Hum, Raluca Maracineanu, Laura Atyim, Daniela Gurgus, Robert-Nikolas Folescu, Loredana Gabriela Stana, Roxana Folescu

**Affiliations:** 1 Doctoral School, “Victor Babes” University of Medicine and Pharmacy, 300041 Timisoara, Romania; ivona.ursu@umft.ro (I.M.H.); laura.atyim@umft.ro (L.A.); 2 Department of Balneology, Medical Recovery and Rheumatology, Family Medicine University Clinic, Center for Preventive Medicine, “Victor Babes” University of Medicine and Pharmacy, 300041 Timisoara, Romania; gurgus.daniela@umft.ro; 3 Faculty of Medicine, “Victor Babes” University of Medicine and Pharmacy, 300041 Timisoara, Romania; robert.folescu@student.umft.ro; 4 Department I, Discipline of Anatomy and Embryology, “Victor Babes” University of Medicine and Pharmacy, 300041 Timisoara, Romania; loredana.stana@umft.ro; 5 Department of Balneology, Medical Recovery and Rheumatology, Family Medicine University Clinic, Center for Preventive Medicine, Center for Advanced Research in Cardiovascular Pathology and Hemostaseology, “Victor Babes” University of Medicine and Pharmacy, 300041 Timisoara, Romania; folescu.roxana@umft.ro

**Keywords:** MRONJ, antiresorptive therapy, treatment indication, MRONJ severity

## Abstract

**Background/Objectives**: Medication-related osteonecrosis of the jaw (MRONJ) may differ in clinical presentation and severity according to the indication for antiresorptive therapy. This study compared patients with MRONJ treated for an oncologic indication or osteoporosis and evaluated factors associated with advanced disease, defined as American Association of Oral and Maxillofacial Surgeons (AAOMS) stages II and III. **Methods**: This retrospective observational study included 119 patients with MRONJ, comprising 73 patients with an oncologic indication and 46 treated for osteoporosis. Demographic, clinical, treatment-related, and selected biochemical data were collected from medical records. MRONJ severity was classified according to the AAOMS staging system. Between-group differences were assessed using non-parametric and categorical tests, and multivariable logistic regression was used to examine associations with advanced MRONJ after adjustment for selected covariates. **Results**: Patients with an oncologic indication were younger and had a shorter duration of antiresorptive therapy than those treated for osteoporosis. The distribution of AAOMS stages differed between groups (*p* = 0.001). Stage III disease was more frequent in the oncologic group than in the osteoporosis group (32.9% vs. 8.7%), whereas stage II frequencies were similar (43.8% vs. 41.3%). Advanced MRONJ was also more frequent in the oncologic group (76.7% vs. 50.0%; *p* = 0.003). In the multivariable model, older age (OR, 1.16; 95% CI, 1.06–1.27; *p* = 0.001) and diabetes mellitus (OR, 8.88; 95% CI, 2.29–34.50; *p* = 0.002) were associated with higher odds of advanced MRONJ. Compared with oncologic patients, those treated for osteoporosis had lower adjusted odds of advanced disease (OR, 0.14; 95% CI, 0.05–0.44; *p* < 0.001). No statistically significant association between recorded treatment duration and advanced MRONJ was detected in the sensitivity analysis. **Conclusions**: MRONJ severity differed between patients treated for an oncologic indication and those treated for osteoporosis, with the difference particularly pronounced for stage III disease. These findings should be interpreted in the context of differences in antiresorptive treatment regimens and do not establish an independent causal effect of treatment indication.

## 1. Introduction

Medication-related osteonecrosis of the jaw (MRONJ) is a clinically important adverse condition associated primarily with antiresorptive medications, particularly bisphosphonates and denosumab. It predominantly affects the maxillofacial skeleton and may present with exposed or probeable bone, pain, infection, and fistula formation [1,2]. The biological mechanisms underlying MRONJ are complex and remain incompletely understood. Current evidence supports a multifactorial process involving suppression of bone remodeling, local infection and inflammation, impaired angiogenesis, soft-tissue toxicity, and alterations in immune and cellular responses [2,3,4]. The resulting clinical presentation is highly variable, with disease ranging from limited local lesions to extensive necrotic involvement and associated complications [1,3].

The clinical context in which antiresorptive therapy is administered is an important consideration when interpreting the clinical characteristics of MRONJ. In patients with osteoporosis, antiresorptive treatment is primarily intended to reduce the risk of fragility fractures, whereas in patients with advanced malignancies and bone involvement, it is used to reduce skeletal-related complications [2,5,6]. These clinical indications are generally associated with different treatment regimens and exposure patterns, making treatment indication an important consideration when comparing patients with osteoporosis and those treated for an oncologic indication.

The development of MRONJ is also influenced by a range of local and systemic factors that may interact with antiresorptive exposure [7]. Local dental conditions, particularly tooth extraction, periodontal disease, periapical infection, and other inflammatory processes involving the jaws, have been frequently associated with MRONJ [8,9,10]. Importantly, the presence of a dental trigger does not necessarily imply a direct causal relationship, as pre-existing infection or inflammation may also contribute to the development of MRONJ [8,9]. Patient-related factors, including advanced age, smoking, renal impairment, and other systemic comorbidities, have also been investigated in relation to MRONJ susceptibility and clinical presentation [8]. Diabetes mellitus has also been proposed as an important systemic risk factor, potentially through effects on bone remodeling, microvascular function, and immune responses [11]. In patients receiving antiresorptive therapy for an oncologic indication, concomitant treatments such as chemotherapy, corticosteroids, and antiangiogenic agents may further modify the risk profile of MRONJ [12,13]. The contribution of these factors may therefore extend beyond MRONJ development and may also be relevant to the clinical severity observed once the condition has been established.

Previous comparative studies have suggested that the clinical phenotype of MRONJ may differ according to the underlying indication for antiresorptive therapy. Patients treated for osteoporosis and cancer have been reported to differ in selected clinical characteristics [5,14]. In the study by Feng et al., patients treated for cancer developed MRONJ after a shorter interval from initiation of antiresorptive therapy than patients with osteoporosis, while the two groups also differed in treatment characteristics and the use of concomitant antiangiogenic therapy [14]. More recently, Shin et al. reported differences in clinical findings between oncologic and osteoporotic patients [5]. Together, these findings indicate that MRONJ may present differently according to the underlying clinical indication for antiresorptive therapy.

The clinical severity of MRONJ is heterogeneous and may range from nonspecific clinical findings to extensive necrotic lesions associated with infection and other complications. The AAOMS classification distinguishes stages according to the extent of bone involvement and associated clinical findings, with stages I–III representing increasing clinical severity and extent of disease [1]. Stage II is characterized by exposed and necrotic bone associated with pain and infection, whereas stage III includes more extensive disease with complications such as pathological fracture, extraoral fistula, or extension beyond the alveolar bone [1]. Assessment of the MRONJ stage at diagnosis therefore provides an important framework for characterizing disease severity and comparing clinical phenotypes across patient populations.

Despite the available comparative evidence, the factors underlying differences in MRONJ presentation and severity between patients receiving antiresorptive therapy for osteoporosis and those receiving treatment for an oncologic indication remain incompletely characterized. Existing studies have identified differences between these patient populations, but such differences may reflect several interrelated factors, including antiresorptive treatment characteristics, local precipitating conditions, systemic comorbidities, and concomitant medications [8,12,13]. The substantial variability in clinical characteristics reported among patients with MRONJ further supports the need for comprehensive characterization of affected patients [15]. Therefore, the present retrospective study aimed to compare the demographic, treatment-related, clinical, and biochemical characteristics of patients with MRONJ according to the underlying indication for antiresorptive therapy, namely osteoporosis or an oncologic indication. Particular attention was given to antiresorptive treatment characteristics and duration, MRONJ stage and anatomical location, local precipitating factors, relevant comorbidities, and concomitant medications. In addition, associations with advanced MRONJ, defined as AAOMS stages II–III, were examined using multivariable logistic regression adjusted for the selected covariates.

## 2. Materials and Methods

### 2.1. Study Design and Patient Population

This retrospective observational study included consecutive patients with MRONJ identified from the medical records of the Department of Oral and Maxillofacial Surgery, Municipal Emergency Hospital, affiliated with the Victor Babeș University of Medicine and Pharmacy, Timișoara, Romania. The MRONJ case list was reviewed to identify patients diagnosed at our center between November 2024 and September 2026, after which their medical records were assessed for eligibility and data availability. Thus, the study period refers to the date of MRONJ diagnosis at our center, rather than the date of antiresorptive treatment initiation or hospital admission.

Patients were eligible for inclusion if they had a documented diagnosis of MRONJ associated with antiresorptive therapy, had received antiresorptive treatment for either an oncologic indication or osteoporosis, and had sufficient clinical and treatment-related information to classify the treatment indication and MRONJ severity.

A total of 135 patients were identified. Patients were excluded when essential information required for the present analysis was unavailable. Specifically, 2 patients were excluded because the indication for antiresorptive treatment could not be established, 8 patients were excluded because information regarding antiresorptive treatment was insufficient, and 6 patients were excluded because the AAOMS stage of MRONJ was not available. Thus, the final study cohort comprised 119 patients with MRONJ, including 73 patients with an oncologic indication for antiresorptive therapy and 46 patients treated for osteoporosis. Baseline vitamin D measurements were not an eligibility requirement but were available for all 119 included patients. Basic data were not systematically retained for the excluded patients; therefore, a descriptive comparison between excluded and included patients could not be performed.

A subset of 72 patients included in the present cohort had previously been reported in a retrospective study investigating serum vitamin D levels and clinical characteristics of patients with MRONJ according to antiresorptive treatment group and lesion location [16]. The present study expands the cohort to 119 patients, including 47 additional patients identified during an extended study period. Although some clinical variables, including serum vitamin D levels, demographic characteristics, comorbidities, lesion location, and AAOMS stage, overlap with those examined in the previous publication, the present study addresses a distinct research question, focusing on MRONJ severity according to treatment indication (oncologic indication versus osteoporosis) and factors associated with advanced disease. The current analyses include multivariable logistic regression and sensitivity analyses designed to investigate factors associated with disease severity.

### 2.2. Data Collection and Variables

Clinical, demographic, and treatment-related information was retrospectively retrieved from the patients’ medical records. Demographic variables included age and sex. Clinical characteristics comprised body mass index (BMI), smoking status, diabetes mellitus, cardiovascular disease, thyroid disease, rheumatoid arthritis, and renal disease. Information on concomitant medication use was also collected, including antidiabetic, antihypertensive, anticoagulant, corticosteroid, and antithyroid medications. Antithyroid medication is referred specifically to medication used to treat hyperthyroidism. Corticosteroid use was recorded based on medication documented in the medical records at the time of MRONJ assessment. Previous corticosteroid exposure was not systematically assessed. Data on chemotherapy and antiangiogenic treatments were not available for analysis.

Smoking status was extracted from the medical records at the time of MRONJ assessment. Current and former smokers were grouped into a single category. The records did not permit a standardized assessment of smoking intensity, cumulative exposure, or time since smoking cessation. Diabetes mellitus was identified based on the diagnosis documented in the medical records and was present before the diagnosis of MRONJ. Blood glucose measurements were also recorded; however, glycemic control was not assessed using a predefined criterion for this study.

Treatment-related data included the underlying indication for antiresorptive therapy, specific antiresorptive agent, route of administration, dose, administration frequency, and treatment duration. Patients were categorized according to whether antiresorptive treatment had been administered for an oncologic indication or osteoporosis. Treatment duration was calculated in months as the elapsed time from initiation of antiresorptive therapy to the diagnosis of MRONJ. Treatment interruptions, discontinuation before diagnosis, and switches between antiresorptive agents were not systematically characterized in the available medical records. Therefore, treatment duration represents recorded elapsed time rather than a standardized measure of cumulative drug exposure and may not be directly comparable across different treatment regimens.

Serum 25-hydroxyvitamin D concentrations (µg/L) were obtained from laboratory measurements performed during the hospital assessment, before any intervention related to MRONJ management. Vitamin D levels were determined using an electrochemiluminescence immunoassay.

In patients receiving antiresorptive therapy for an oncologic indication, the presence of bone metastases was additionally documented as a disease-related characteristic.

### 2.3. MRONJ Diagnosis and Staging

The diagnosis of MRONJ was established according to the 2022 Position Paper of the AAOMS [1]. The diagnostic assessment included current or previous exposure to an antiresorptive agent, the presence of exposed bone or bone that could be probed through an intraoral or extraoral fistula in the maxillofacial region persisting for more than 8 weeks, and the absence of previous radiation therapy to the jaws or metastatic disease involving the jaws. MRONJ severity was classified according to the AAOMS staging system. No cases classified as AAOMS stage 0 were identified in this cohort; all 119 patients were classified as having stage I, II, or III MRONJ. For the primary analysis, AAOMS stages II and III were considered advanced MRONJ, whereas stage I served as the reference category. This study-specific grouping was used to distinguish patients with stage I disease from those with stage II or III disease.

For the purposes of this study, the MRONJ stage was based on the stage documented by the treating clinician following the patient’s first assessment at our center, where all patients in the cohort were diagnosed. According to the available medical records, none of the patients had received treatment for MRONJ before this assessment. The documented stage was retrospectively extracted from the clinical records. No independent reassessment of staging was performed specifically for this study. Although imaging investigations had been performed as part of routine clinical care, the original imaging data were not available to the study investigators for independent review, and the original clinical findings were not systematically re-evaluated. No formal adjudication procedure was used for uncertain or conflicting findings.

The unit of analysis was the individual patient, with one observation included per patient. Anatomical location was recorded as either mandibular or maxillary involvement; no patients with involvement of both jaws were identified in the study cohort. The available records did not permit a standardized retrospective assessment of how multiple lesions, recurrent episodes, or repeat presentations were handled.

The relationship between tooth extraction and MRONJ was assessed retrospectively based on the clinical information documented in the medical records. Cases were classified as extraction-related when the records indicated that MRONJ developed following tooth extraction performed because of periodontal disease, periapical infection, surgical indications, or a non-restorable tooth. No predefined time interval between extraction and MRONJ diagnosis was applied. Non-extraction-related MRONJ included cases associated with dental implant placement, denture-related trauma, dentoalveolar surgery without tooth extraction, local infection or periodontal disease without extraction, local trauma, or cases occurring spontaneously without an identifiable local precipitating factor. The retrospective records did not always allow reliable determination of whether osteonecrosis was already developing before tooth extraction.

### 2.4. Statistical Analysis

Statistical analyses were performed using JASP software, version 0.98.1 (JASP Team, University of Amsterdam, Amsterdam, The Netherlands; https://jasp-stats.org (accessed on 29 September 2026)). Additional analyses were performed using Python 3.13.5 (Python Software Foundation, Wilmington, DE, SUA; https://www.python.org (accessed on 15 Octomber 2026)). Firth’s penalized-likelihood logistic regression was implemented in Python using Jeffreys-prior penalization, with numerical optimization performed using SciPy. Continuous variables are presented as median (IQR), whereas categorical variables are presented as frequencies and percentages. Between-group comparisons of continuous variables were performed using the Mann–Whitney U test, and categorical variables were compared using Pearson’s chi-square test or Fisher’s exact test, as appropriate.

Differences in treatment duration across AAOMS stages I, II, and III were assessed using the Kruskal–Wallis test. When the overall test was significant, pairwise comparisons were performed using Dunn’s post hoc test with Bonferroni correction. The effect size for the Kruskal–Wallis test was expressed as rank *ε*^2^.

To examine associations with advanced MRONJ after adjustment for the selected covariates, a multivariable logistic regression model was constructed with advanced MRONJ, defined as AAOMS stages II–III versus stage I, as the dependent variable. Candidate covariates were selected a priori based on clinical judgment and their perceived relevance to disease severity. The model included age, serum vitamin D concentration, treatment indication (osteoporosis vs. oncologic), smoking status, diabetes mellitus, anatomical location (maxilla vs. mandible), and extraction-related MRONJ. The linearity of the relationships between continuous predictors and the log odds of the outcome was assessed by adding centered quadratic terms for age and serum vitamin D concentration to the primary logistic regression model, and a centered quadratic term for age to the secondary stage III model. The statistical significance of the quadratic terms and model fit criteria, including the Akaike information criterion (AIC) and Bayesian information criterion (BIC), were examined. Adjusted odds ratios (ORs) with 95% confidence intervals (CIs) were estimated for each predictor. ORs for continuous predictors represent a one-unit increase in the corresponding variable.

Model performance was assessed using the likelihood-ratio chi-square test, Nagelkerke’s *R*^2^, the area under the receiver operating characteristic curve (AUC), and the Brier score. Internal validation was performed using 1000 bootstrap resamples. For each resample, model performance was evaluated in both the bootstrap sample and the original dataset, and the difference between these estimates was used to quantify optimism. For the AUC and Brier score, mean optimism was subtracted from the apparent performance. The calibration intercept and slope were corrected by subtracting their respective mean optimism estimates from their ideal values of 0 and 1, respectively. Firth’s penalized-likelihood logistic regression was performed as a sensitivity analysis using the same outcome and covariates as in the primary model to assess the robustness of the estimates in the presence of sparse data, particularly for diabetes mellitus. Multicollinearity among predictors was assessed using variance inflation factors (VIFs).

To assess the potential influence of sex, two sensitivity analyses were performed. First, sex was added to the primary multivariable logistic regression model. Second, a separate parsimonious model was fitted among women only, including age, serum vitamin D concentration, treatment indication, and diabetes mellitus as covariates. The direction, magnitude, and 95% CIs of the adjusted ORs for treatment indication were examined in both sensitivity analyses. The distribution of advanced MRONJ (AAOMS stages II–III) and stage III disease was described across the three observed sex-by-indication subgroups: men receiving antiresorptive therapy for an oncologic indication, women receiving therapy for an oncologic indication, and women treated for osteoporosis.

Treatment indication was included in the multivariable model as a categorical variable distinguishing patients receiving antiresorptive therapy for an oncologic indication from those treated for osteoporosis. In this cohort, treatment indication was strongly linked to the antiresorptive agent, route of administration, dose, and administration frequency. These treatment characteristics were therefore not entered simultaneously into the model because their effects could not be reliably distinguished given the absence of overlap in treatment regimens between groups. Accordingly, the adjusted association with treatment indication was interpreted as reflecting the broader clinical treatment context rather than an isolated effect of the underlying indication. A sensitivity analysis was also performed by additionally including treatment duration in the multivariable model.

An exploratory logistic regression analysis was performed for stage III MRONJ versus stages I–II using the same seven covariates as in the primary model. Model convergence was assessed, and influence diagnostics were used to identify potentially influential observations. Leave-one-out analyses were conducted by successively excluding individual patients and refitting the model to assess the stability of the association between extraction-related MRONJ and stage III disease. Firth’s penalized-likelihood logistic regression was performed using the same covariates to assess the robustness of the secondary model estimates, with profile penalized-likelihood confidence intervals and *p*-values. The functional form of age was evaluated by adding a centered quadratic term and comparing model fit using the AIC.

All statistical tests were two-sided, and a *p*-value < 0.05 was considered statistically significant. No missing data were present for the variables included in the final analyses; therefore, all 119 patients were included in the primary multivariable logistic regression model.

### 2.5. Ethics Statement

The study complied with the Declaration of Helsinki and the principles of good clinical research practice. The study protocol was approved by the Ethics Committee for Scientific Research of Victor Babeș University of Medicine and Pharmacy, Timișoara (No. 106/11 October 2024). Written informed consent was obtained from the participants for study participation and for the use and publication of study data, with confidentiality maintained throughout the study.

ChatGPT, version 4.0 (OpenAI, San Francisco, CA, USA) was used to assist with language refinement and improve the clarity and readability of the manuscript. The authors reviewed and edited the output and take full responsibility for the final manuscript.

## 3. Results

A total of 119 patients with MRONJ were included, comprising 73 patients with an oncologic indication for antiresorptive therapy and 46 patients treated for osteoporosis. Patients in the osteoporotic group were older than those in the oncologic group (median age, 70 [IQR, 68–73] vs. 66 [IQR, 59–69] years; *p* < 0.001). The proportion of female patients was higher in the osteoporotic group (100% vs. 53.4%; *p* < 0.001). No significant between-group differences were observed for smoking, BMI, diabetes mellitus, or cardiovascular disease (all *p* > 0.05). Thyroid disease was more frequent among osteoporotic patients than among oncologic patients (19.6% vs. 1.4%; *p* < 0.001). Rheumatoid arthritis and renal disease were rare and did not differ significantly between groups. Serum vitamin D concentrations were lower in the osteoporotic group than in the oncologic group (median, 25.42 [IQR, 21.05–28.57] vs. 27.59 [IQR, 23.44–31.29] µg/L; *p* = 0.035) (Table 1).

Concomitant medication use was generally similar between the two groups, except for antithyroid medication, which was more frequently recorded in the osteoporotic group than in the oncologic group (19.6% vs. 1.4%; *p* < 0.001). No significant differences were observed for antidiabetic, antihypertensive, anticoagulant, or corticosteroid medication (all *p* > 0.05) (Table 2).

The distribution of malignancies and the presence of bone metastases among patients receiving antiresorptive therapy for an oncologic indication are summarized in Table 3.

Antiresorptive treatment characteristics differed according to treatment indication (Table 4). Patients in the oncologic group received zoledronic acid or denosumab, whereas those in the osteoporotic group received risedronate, alendronate, or ibandronate. In the oncologic group, treatment was administered intravenously or subcutaneously, at 3- or 4-week intervals, whereas all osteoporotic patients received oral treatment administered weekly or monthly. Treatment duration was significantly shorter in the oncologic group than in the osteoporotic group (median, 11 [IQR, 9–14] vs. 42 [IQR, 34–47] months; *p* < 0.001).

Within the oncologic group, AAOMS stage distribution was examined according to antiresorptive agent. Among patients receiving zoledronic acid (*n* = 42), 10 had stage I, 16 had stage II, and 16 had stage III MRONJ. Among patients receiving denosumab (*n* = 31), 7 had stage I, 16 had stage II, and 8 had stage III MRONJ. The distribution of AAOMS stages did not differ significantly between the two groups (*p* = 0.455). This exploratory comparison was not designed to establish drug-specific effects, and the findings should be interpreted cautiously given the limited subgroup sizes and potential differences in clinical characteristics.

Clinical characteristics and MRONJ severity according to treatment indication are presented in Table 5. Mandibular involvement was more frequent among patients with an oncologic indication than among those treated for osteoporosis (67.1% vs. 47.8%; *p* = 0.037). The distribution of AAOMS stages differed between the two groups (*p* = 0.001). Stage I disease was more frequent in the osteoporosis group than in the oncologic group (50.0% vs. 23.3%), whereas stage III disease was more frequent in the oncologic group (32.9% vs. 8.7%). Stage II frequencies were similar between the oncologic and osteoporosis groups (43.8% vs. 41.3%). When stages II and III were combined, advanced MRONJ was more frequent in the oncologic group than in the osteoporosis group (76.7% [56/73] vs. 50.0% [23/46]; *p* = 0.003), corresponding to an absolute difference of 26.7 percentage points (95% CI, 9.1–42.8). The crude odds ratio was 3.29 (95% CI, 1.49–7.28). The frequency of extraction-related MRONJ was similar between the two groups (65.8% vs. 69.6%; *p* = 0.666).

In an exploratory analysis, antiresorptive treatment duration differed significantly across AAOMS stages (Kruskal–Wallis *H* = 6.36, *df* = 2; *p* = 0.042; rank *ε*^2^ = 0.054). Post hoc analysis showed a significant difference between stages I and III (*p* = 0.037), whereas comparisons between stages I and II (*p* = 1.000) and stages II and III (*p* = 0.212) were not significant (Table 6).

The observed decrease in median antiresorptive treatment duration across AAOMS stages should be interpreted in the context of the differences between treatment-indication groups. In the oncologic group, median treatment duration was 10 months (IQR, 8–12) for stage I, 12 months (IQR, 10.75–15) for stage II, and 11 months (IQR, 9.75–13.5) for stage III. In the osteoporosis group, the corresponding medians were 42 months (IQR, 33–45.5), 42 months (IQR, 34–46.5), and 50.5 months (IQR, 46–59), respectively. Only four patients in the osteoporosis group had stage III disease. Thus, treatment duration did not decrease consistently with increasing AAOMS stage within either indication group. The pooled pattern may partly reflect differences in group composition rather than a within-group association between treatment duration and MRONJ severity.

In univariable logistic regression analyses, older age was associated with higher odds of advanced MRONJ (OR, 1.08; 95% CI, 1.02–1.13; *p* = 0.006). Treatment for osteoporosis was associated with lower odds of advanced disease compared with an oncologic indication (OR, 0.30; 95% CI, 0.14–0.67; *p* = 0.003). Diabetes mellitus was associated with higher odds of advanced MRONJ (OR, 10.87; 95% CI, 3.09–38.18; *p* < 0.001). Serum vitamin D concentration, smoking, anatomical location, and extraction-related MRONJ were not significantly associated with advanced disease in univariable analyses (all *p* > 0.05) (Table 7).

To identify factors associated with advanced MRONJ, a multivariable logistic regression model was constructed with AAOMS stages II–III versus stage I as the dependent variable. The model was statistically significant (likelihood ratio *χ*^2^ = 46.46, *p* < 0.001; Nagelkerke *R*^2^ = 0.448). No relevant multicollinearity was identified among the model predictors, with VIF values ranging from 1.02 to 2.11.

The multivariable model had an apparent AUC of 0.849 (95% bootstrap CI, 0.767–0.919). Internal validation using 1000 bootstrap resamples yielded an optimism-corrected AUC of 0.811 and an optimism-corrected Brier score of 0.166. The optimism-corrected calibration intercept was 0.097, and the optimism-corrected calibration slope was 0.774. These findings suggest some degree of overfitting and should be interpreted cautiously, as internal validation does not establish external predictive validity.

Older age was associated with higher odds of advanced MRONJ (OR, 1.16; 95% CI, 1.06–1.27; *p* = 0.001). Compared with the oncologic group, patients treated for osteoporosis had lower adjusted odds of advanced MRONJ (OR, 0.14; 95% CI, 0.05–0.44; *p* < 0.001). Diabetes mellitus was also associated with advanced MRONJ (OR, 8.88; 95% CI, 2.29–34.50; *p* = 0.002). Among patients with diabetes mellitus, 37 of 40 had advanced MRONJ and 3 had stage I disease. Among patients without diabetes, 42 of 79 had advanced MRONJ, and 37 had stage I disease. The small number of patients with diabetes in the non-advanced group warrants cautious interpretation of the magnitude and precision of the adjusted association. Serum vitamin D, smoking, anatomical location, and extraction-related MRONJ were not significantly associated with advanced disease (all *p* > 0.05). Detailed results are presented in Table 8. In the assessment of functional form, neither the centered quadratic term for age (*p* = 0.505) nor that for serum vitamin D concentration (*p* = 0.601) was statistically significant. The model including quadratic terms had higher AIC and BIC values than the corresponding linear model, providing no evidence of improved model fit.

In a sensitivity analysis using Firth’s penalized-likelihood logistic regression, older age remained associated with higher odds of advanced MRONJ (OR, 1.145; 95% CI, 1.058–1.255; *p* = 0.002). Treatment for osteoporosis was associated with lower odds of advanced MRONJ compared with an oncologic indication (OR, 0.171; 95% CI, 0.056–0.469; *p* = 0.001). Diabetes mellitus also remained associated with advanced MRONJ (OR, 6.873; 95% CI, 2.204–28.243; *p* = 0.002). The remaining covariates were not significantly associated with advanced MRONJ. Overall, the findings were consistent with those of the primary multivariable logistic regression model. The diabetes mellitus estimate should be interpreted cautiously because only three patients with diabetes had non-advanced MRONJ.

**Figure 1:**
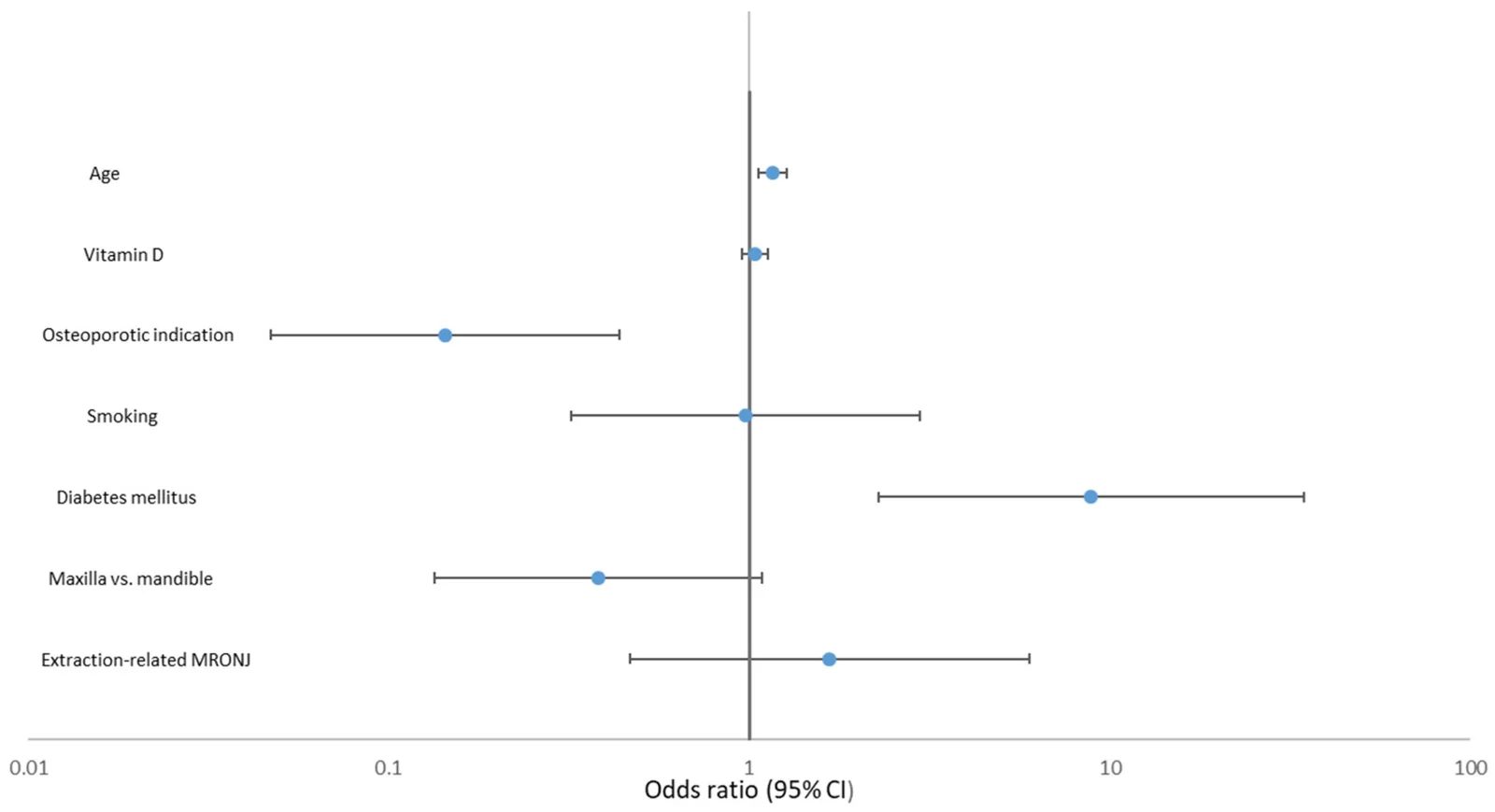
Forest plot showing ORs and 95% CIs for factors associated with advanced MRONJ. Blue circles represent the point estimates of the ORs, and horizontal error bars represent 95% CIs. The vertical reference line indicates an OR of 1. Age ORs are expressed per 1-year increase in age, and vitamin D ORs are expressed per 1 µg/L increase in serum vitamin D concentration. The *x*-axis uses a logarithmic scale.

The estimated ORs and corresponding 95% CIs are illustrated in Figure 1.

In a sensitivity analysis additionally adjusted for antiresorptive treatment duration, the association between treatment indication and advanced MRONJ remained statistically significant. Compared with the oncologic group, patients treated for osteoporosis had lower adjusted odds of advanced MRONJ (OR, 0.12; 95% CI, 0.02–0.86; *p* = 0.035). Treatment duration was not significantly associated with advanced MRONJ (OR per month, 1.01; 95% CI, 0.95–1.06; *p* = 0.836). Older age remained associated with higher odds of advanced disease (OR per year, 1.16; 95% CI, 1.06–1.27; *p* = 0.001), as did diabetes mellitus (OR, 8.89; 95% CI, 2.29–34.53; *p* = 0.002). Serum vitamin D, smoking, anatomical location, and extraction-related MRONJ were not significantly associated with advanced disease (all *p* > 0.05) (Table 9). The model was statistically significant (likelihood-ratio *χ*^2^ = 46.50, *p* < 0.001; Nagelkerke *R*^2^ = 0.449). The VIFs were highest for treatment duration (4.24) and treatment indication (4.30), indicating that the relationship between these variables should be considered when interpreting the adjusted estimates.

The distribution of MRONJ severity was examined across the three sex-by-indication subgroups. Advanced MRONJ (AAOMS stages II–III) was observed in 26 of 34 men in the oncologic group (76.5%), 30 of 39 women in the oncologic group (76.9%), and 23 of 46 women in the osteoporosis group (50.0%). Stage III disease occurred in 15 men in the oncologic group (44.1%), 9 women in the oncologic group (23.1%), and 4 women in the osteoporosis group (8.7%).

To assess the potential influence of sex imbalance on the observed association between treatment indication and advanced MRONJ, two sensitivity analyses were performed. First, sex was added to the multivariable logistic regression model in the full cohort. The association between osteoporosis treatment and lower odds of advanced MRONJ remained similar in direction and magnitude (OR, 0.087; 95% CI, 0.022–0.352; *p* < 0.001), whereas sex was not significantly associated with advanced disease (male vs. female: OR, 0.38; 95% CI, 0.092–1.605; *p* = 0.190). Second, a parsimonious model including women only (*n* = 85; 39 patients with an oncologic indication and 46 treated for osteoporosis) showed a similar association (osteoporosis vs. oncologic indication: OR, 0.100; 95% CI, 0.024–0.410; *p* = 0.001). In this subgroup, older age was associated with higher odds of advanced MRONJ (OR, 1.13; 95% CI, 1.040–1.228; *p* = 0.004), as was diabetes mellitus (OR, 13.46; 95% CI, 2.549–71.061; *p* = 0.002), although this estimate had a wide confidence interval. These analyses do not eliminate potential confounding by differences in antiresorptive agents and treatment regimens.

In a secondary multivariable analysis of stage III versus stages I–II, older age was associated with higher odds of stage III disease (OR, 1.21; 95% CI, 1.07–1.37; *p* = 0.002), whereas patients with an osteoporotic indication had lower odds of stage III disease than those with an oncologic indication (OR, 0.08; 95% CI, 0.02–0.32; *p* < 0.001). Extraction-related MRONJ was also associated with higher odds of stage III disease (OR, 3.92; 95% CI, 1.01–15.20; *p* = 0.048). Serum vitamin D, smoking, diabetes mellitus, and anatomical location were not significantly associated with stage III disease (all *p* > 0.05). The analysis included all 119 patients, of whom 28 had stage III disease and 91 had stages I–II. Given the limited number of stage III cases and the wide confidence interval for extraction-related MRONJ, these findings should be interpreted cautiously. The overall model was statistically significant (likelihood-ratio *χ*^2^ = 28.998, *p* < 0.001; Nagelkerke *R*^2^ = 0.326). Full results for all seven predictors, including nonsignificant associations, are presented in Table 10.

The centered quadratic term for age was not statistically significant (*p* = 0.746), and the model including this term had a higher AIC than the corresponding linear model. Thus, the analysis did not provide evidence that adding a quadratic age term improved model fit.

The standard logistic regression model converged without convergence warnings, although influence diagnostics identified potentially influential observations (maximum Cook’s distance, 0.111). In leave-one-out analyses, the adjusted OR for extraction-related MRONJ ranged from 3.43 to 5.24, and the association was no longer statistically significant in 49 of the 119 analyses, indicating sensitivity to individual observations.

In the Firth penalized-likelihood sensitivity analysis, older age remained associated with higher odds of stage III disease (OR, 1.18; 95% profile penalized-likelihood CI, 1.07–1.34; *p* < 0.001), whereas an osteoporotic indication was associated with lower odds than an oncologic indication (OR, 0.11; 95% profile penalized-likelihood CI, 0.03–0.35; *p* < 0.001). The association with extraction-related MRONJ remained positive but did not reach conventional statistical significance (OR, 3.30; 95% profile penalized-likelihood CI, 0.97–12.65; *p* = 0.057). The other covariates were not significantly associated with stage III disease.

## 4. Discussion

MRONJ has a multifactorial clinical presentation, influenced by the type of antiresorptive therapy, the underlying disease, and local and systemic patient characteristics [1,2]. Disease severity ranges from limited jaw involvement to more extensive lesions, with the AAOMS staging system providing a standardized approach to clinical classification [1]. These differences are particularly relevant when patients receiving antiresorptive therapy for osteoporosis are compared with those treated for an oncologic indication, as the therapeutic context and underlying disease differ between these groups [2].

In our cohort, patients treated for an oncologic indication more frequently presented with advanced MRONJ than those treated for osteoporosis (76.7% vs. 50.0%). However, the difference in stage distribution was particularly pronounced for stage III disease, which occurred in 32.9% of oncologic patients compared with 8.7% of osteoporotic patients, whereas stage II frequencies were similar between groups (43.8% vs. 41.3%). This pattern suggests that the difference in disease severity between the groups was driven predominantly by the higher proportion of stage III cases in the oncologic group. Notably, this difference was observed despite the substantially longer antiresorptive treatment duration in the osteoporotic group.

An important consideration in interpreting the present findings is the close relationship between treatment indication and antiresorptive regimen. Patients treated for an oncologic indication received intravenous zoledronic acid or subcutaneous denosumab, whereas patients treated for osteoporosis received oral bisphosphonates. These groups therefore differed not only in the underlying clinical indication but also in the antiresorptive agent, route of administration, dose, and administration frequency. Consequently, the observed association between treatment indication and MRONJ severity cannot be attributed to the indication itself independently of the associated treatment regimen and clinical context. Although the association persisted after adjustment for the selected patient-related and local clinical characteristics, residual confounding by treatment regimen and other indication-related factors cannot be excluded. Accordingly, treatment indication should be interpreted as a marker of the broader clinical context and associated antiresorptive regimen, rather than as an independent exposure whose effect can be separated from these treatment characteristics.

Patients treated for an oncologic indication may also differ from those treated for osteoporosis in the presence of metastatic disease, systemic anticancer treatment, and other concomitant medications [2]. Previous evidence has reported a higher occurrence of MRONJ among patients with malignant disease than among those treated for benign conditions, although these studies primarily address MRONJ development rather than severity among patients with established disease [6]. These factors should therefore be considered when interpreting the observed differences in MRONJ severity.

In the present study, diabetes mellitus was associated with higher adjusted odds of advanced MRONJ, consistent with previous evidence linking diabetes to the development of MRONJ. Tani et al. reported an association between diabetes and the development of stage ≥ 2 MRONJ during follow-up of patients with prostate cancer receiving bone-modifying agents [17]. This provides related contextual evidence, although it differs from our comparison of severity among patients with established MRONJ. The MASCC/ISOO/ASCO clinical practice guideline identifies uncontrolled diabetes among systemic conditions that may increase susceptibility to MRONJ [18]. A recent systematic review and meta-analysis also reported a higher prevalence of bisphosphonate-associated osteonecrosis among patients with diabetes, although the certainty of the available evidence was considered very low [19]. Several mechanisms may plausibly underlie this association, including microvascular impairment, endothelial dysfunction, altered bone remodeling, chronic inflammation, and impaired immune responses [11]. Our findings further suggest that diabetes may be associated with MRONJ severity among patients with established disease. However, this association should be interpreted cautiously. Although diabetes mellitus remained associated with advanced MRONJ in the Firth penalized-likelihood sensitivity analysis, only three of the 40 patients with diabetes had non-advanced disease. This sparse-data pattern may affect the stability and precision of the estimated association, and penalized regression does not eliminate the uncertainty arising from the limited number of patients in this subgroup. Given the retrospective observational design, these findings do not establish a causal relationship.

Older age was associated with higher adjusted odds of advanced MRONJ in our analysis, with increasing age corresponding to higher odds of AAOMS stage II–III disease. This finding is consistent with the results of Nashi et al., who reported that older age was independently associated with stage ≥ 2 antiresorptive agent-related osteonecrosis of the jaw in a retrospective cohort of 143 patients [20]. However, the relationship between age and MRONJ severity has not been consistent across studies. Feng et al., in a smaller cohort focused specifically on stage III disease, reported that age ≤ 65 years was associated with more severe MRONJ, illustrating that the association between age and disease severity may vary according to the clinical characteristics of the population studied [21]. These differences may reflect variations in patient characteristics, underlying disease, antiresorptive regimens, and concomitant treatments. In our cohort, the association between age and advanced disease remained statistically significant after adjustment for treatment indication and other selected clinical variables. The assessment of functional form did not provide evidence that adding quadratic terms for age or serum vitamin D improved model fit. This finding supports retaining linear terms for these predictors in the present analyses, although it does not establish that their relationships with the log odds of MRONJ severity are strictly linear across the entire range of observed values. Accordingly, the estimated odds ratios should be interpreted per one-year increase in age and per one-unit increase in serum vitamin D concentration, under the assumed linear relationships on the logit scale.

Local clinical characteristics were also considered in relation to disease severity. In our cohort, mandibular involvement was more frequent among patients treated for an oncologic indication than among those treated for osteoporosis (67.1% vs. 47.8%; *p* = 0.037). The recorded frequency of extraction-related MRONJ was similar between the two groups (65.8% vs. 69.6%; *p* = 0.666). Tooth extraction is frequently reported in patients with MRONJ and may represent a local precipitating event in susceptible patients, particularly in the presence of pre-existing periodontal or periapical disease [1,22]. However, extraction may also be performed because of dental disease associated with an MRONJ lesion that is already evolving, making the temporal relationship difficult to establish. A recent review has emphasized the interaction between local inflammation, tissue injury, impaired bone remodeling, and altered healing responses in the pathogenesis of MRONJ [22]. In our cohort, the retrospective classification did not always allow reliable distinction between lesions developing after extraction and lesions that may already have been evolving before the procedure. Furthermore, the similar recorded frequencies do not establish that the underlying dental conditions were comparable between groups, as the indications for extraction and the severity of pre-existing dental infection were not systematically assessed. Therefore, the observed similarity in extraction-related classification does not exclude a contribution from local dental disease, inflammation, or other dental factors to MRONJ severity.

Previous studies have also reported differences in MRONJ stage distribution between patients with malignancy and those treated for osteoporosis, although the patterns have not been consistent across cohorts. Feng et al. reported differences in antiresorptive exposure and concomitant antiangiogenic treatment between patients with malignancy and osteoporosis, while Walton et al. found stage II disease to be more frequent among osteoporotic patients and stage I disease more common in the oncologic group [14,23]. These variations across studies may reflect differences in patient characteristics, underlying disease, antiresorptive regimens, and concomitant treatments. Our findings are consistent with a higher proportion of stage III MRONJ in the oncologic group, while stage II frequencies were similar between groups.

The secondary analysis of stage III MRONJ should be interpreted cautiously because only 28 patients had stage III disease. Although extraction-related MRONJ was associated with higher odds of stage III disease in the standard logistic regression model (OR, 3.92; 95% CI, 1.01–15.20; *p* = 0.048), this estimate was imprecise. Sensitivity analyses further indicated that this association was not robust to alternative modeling approaches: the association remained positive but did not reach conventional statistical significance in the Firth penalized-likelihood analysis, and its statistical significance varied in leave-one-out analyses. Taken together, these findings suggest that the observed association may be sensitive to individual observations and should be considered exploratory rather than conclusive. Further studies involving larger, independent cohorts are needed to determine whether this association is reproducible.

The relationship between antiresorptive treatment duration and MRONJ severity remains difficult to define. In the present cohort, treatment duration was substantially longer among patients treated for osteoporosis than among those treated for an oncologic indication. The pooled decrease in median treatment duration across AAOMS stages appears to be influenced by the marked differences between indication groups. In the indication-specific descriptive analysis, treatment duration did not decrease consistently with increasing stage in either group; however, the stage III osteoporosis subgroup included only four patients, limiting interpretation. Previous evidence has mainly addressed treatment duration in relation to the development of MRONJ rather than the severity of established disease. In a systematic review of osteoporotic patients, Aljohani et al. identified treatment duration among the factors associated with MRONJ development [24]. In the adjusted sensitivity model, no statistically significant association between recorded treatment duration and advanced MRONJ was detected (OR per month, 1.01; 95% CI, 0.95–1.06; *p* = 0.836). After adjustment for treatment duration, the association between treatment indication and advanced MRONJ remained statistically significant, but its 95% confidence interval widened from 0.05–0.44 to 0.02–0.86, indicating reduced precision. Therefore, the persistence of statistical significance should be interpreted alongside the increased uncertainty of the estimate. The absence of a statistically significant association with recorded treatment duration does not establish that duration is unrelated to disease severity. Furthermore, treatment duration reflects the elapsed time from treatment initiation to MRONJ diagnosis and may not fully capture cumulative exposure across different treatment regimens. The close relationship between treatment duration, treatment indication, and antiresorptive regimen limits the conclusions that can be drawn regarding the independent contribution of treatment duration to MRONJ severity.

The clinical relevance of identifying patients with more advanced MRONJ extends beyond staging itself, as disease stage may be related to subsequent treatment response and clinical outcomes. Previous studies have reported differences in healing according to disease stage, with less favorable treatment outcomes generally reported in more advanced lesions [25,26]. A recent systematic review and meta-analysis further showed that prognostic factors in MRONJ include medication-related factors, underlying conditions, lesion characteristics, and treatment modalities [27]. Although treatment outcomes were not evaluated in the present study, the higher proportion of advanced and stage III disease among patients treated for an oncologic indication may have implications for subsequent clinical management.

Several strengths should be considered when interpreting these findings. The study specifically evaluated patients with established MRONJ, allowing the analysis to focus on factors associated with disease severity rather than on the development of MRONJ. The comparison of oncologic and osteoporotic indications also allowed treatment context to be considered together with patient-related and local characteristics. In addition, the multivariable analysis incorporated clinically relevant covariates, and sensitivity analyses were performed to assess the stability of the observed associations. Internal validation suggested some degree of model overfitting, indicating that the model’s performance may be lower in other patient populations. Therefore, the model should be considered exploratory, and external validation is needed before clinical application.

Several limitations should be acknowledged. The retrospective observational design limits causal interpretation and may be subject to selection and information bias. The single-center setting, in a specialized oral and maxillofacial surgery department, may have introduced referral bias and limits the generalizability of the findings. Oncologic and osteoporotic patients may differ in referral pathways, access to dental screening, and follow-up practices. Therefore, the higher frequency of stage III MRONJ in the oncologic group may partly reflect patient selection and clinical context rather than differences related to antiresorptive exposure alone. Because referral patterns and access to dental care were not systematically assessed, their contribution could not be quantified. Similarly, symptom duration before diagnosis was not systematically documented, preventing assessment of the potential contribution of diagnostic delay to differences in MRONJ severity at presentation.

A substantial proportion of the cohort (72/119; 60.5%) had been included in a previous study of vitamin D levels and MRONJ characteristics [16]. Although the present study includes 47 additional patients and addresses a distinct research question, some variables and descriptive findings overlap with the previous publication. Accordingly, the findings should be interpreted as an expanded analysis of a partially overlapping cohort rather than as an entirely independent study.

The relatively limited sample size may reduce the precision of estimates, particularly for the less frequent stage III category. In addition, treatment indication was strongly linked to the antiresorptive agent, route of administration, dose, and treatment frequency, preventing meaningful disentanglement of these variables in the multivariable model. Data on chemotherapy and antiangiogenic treatments were unavailable, precluding assessment of their potential contribution to MRONJ severity. Corticosteroid use was assessed from medication documented at the time of MRONJ assessment; previous exposure was not systematically assessed. Therefore, the absence of documented corticosteroid use in the oncologic group should not be interpreted as evidence of no previous exposure, and residual confounding cannot be excluded.

Glycemic control was not assessed using predefined criteria; therefore, the binary diabetes diagnosis could not distinguish between controlled and uncontrolled diabetes, which may have influenced the observed association with advanced MRONJ.

Sensitivity analyses were performed by adding sex to the full-cohort model and by fitting a parsimonious model restricted to women. However, the absence of men in the osteoporosis group limits the assessment of sex-specific effects. The osteoporosis group also consisted exclusively of women receiving oral bisphosphonates; therefore, the findings may not be applicable to men or to patients receiving other antiresorptive regimens for osteoporosis. Treatment duration was evaluated in a sensitivity analysis, but cumulative antiresorptive exposure could not be directly compared across different agents and dosing regimens.

Finally, although MRONJ staging was based on the treating clinicians’ documented assessments according to the AAOMS criteria and imaging investigations performed during clinical care, the original imaging data were unavailable for independent review, and the original clinical findings were not systematically re-evaluated. Therefore, some uncertainty in stage classification cannot be excluded. Treatment outcomes and longitudinal progression were not assessed; consequently, the findings should be interpreted as associations with disease severity at presentation rather than as predictors of subsequent clinical outcomes.

Taken together, our findings indicate that MRONJ severity should be interpreted within the broader clinical context in which antiresorptive therapy is prescribed. The difference between groups was particularly marked for stage III disease, whereas stage II frequencies were similar. The observed associations with treatment indication, age, and diabetes should be interpreted cautiously, given the interrelated treatment characteristics and the limitations of the retrospective study design. These findings do not establish an independent causal effect of treatment indication but suggest that more advanced MRONJ may be encountered more frequently in the oncologic clinical setting.

## 5. Conclusions

In this retrospective cohort, MRONJ severity differed between patients receiving antiresorptive therapy for an oncologic indication and those treated for osteoporosis. The difference was particularly pronounced for stage III disease, which occurred more frequently in the oncologic group, whereas stage II frequencies were similar between groups. This association should be interpreted in the context of the substantial differences in antiresorptive agents, routes of administration, and dosing regimens between groups. The association between diabetes mellitus and advanced MRONJ also warrants cautious interpretation, given the limited number of diabetic patients with non-advanced disease and the resulting uncertainty in the adjusted estimates.

## Figures and Tables

**Table 1:** Demographic, clinical, and biochemical characteristics of patients with MRONJ according to treatment indication.

Variable	Oncologic (*n* = 73)	Osteoporotic (*n* = 46)	*p*-Value
Age (years), median (IQR)	66 (59–69)	70 (68–73)	**<0.001**
Female sex, *n* (%)	39 (53.4%)	46 (100%)	**<0.001**
Smoking, *n* (%)	57 (78.1%)	30 (65.2%)	0.123
BMI (kg/m^2^), median (IQR)	24.9 (22.91–26.08)	25.0 (23.48–27.31)	0.304
Diabetes mellitus, *n* (%)	26 (35.6%)	14 (30.4%)	0.560
Cardiovascular disease, *n* (%)	40 (54.8%)	18 (39.1%)	0.096
Thyroid disease, *n* (%)	1 (1.4%)	9 (19.6%)	**<0.001**
Rheumatoid arthritis, *n* (%)	0 (0%)	1 (2.2%)	0.387
Renal disease, *n* (%)	0 (0%)	1 (2.2%)	0.387
Vitamin D (µg/L), median (IQR)	27.59 (23.44–31.29)	25.42 (21.05–28.57)	**0.035**

Values are presented as median (interquartile range [IQR]) or *n* (%), as appropriate. Statistically significant *p*-values are shown in bold.

**Table 2:** Concomitant medications according to treatment indication.

Concomitant Medication, *n* (%)	Oncologic (*n* = 73)	Osteoporotic (*n* = 46)	*p*-Value
Antidiabetics	26 (35.6)	14 (30.4)	0.560
Antihypertensives	30 (41.1)	19 (41.3)	0.982
Anticoagulants	25 (34.2)	15 (32.6)	0.854
Corticosteroids	0 (0)	1 (2.2)	0.387
Antithyroid medication	1 (1.4)	9 (19.6)	**<0.001**

Values are presented as *n* (%). Statistically significant *p*-values are shown in bold.

**Table 3:** Oncologic characteristics of patients receiving antiresorptive therapy for an oncologic indication (*n* = 73).

Characteristics	*n* (%)
Type of malignancy	
Breast cancer	33 (45.2)
Prostate cancer	23 (31.5)
Bronchopulmonary cancer	8 (11.0)
Multiple myeloma	7 (9.6)
Brain cancer	1 (1.4)
Kidney cancer	1 (1.4)
Bone metastases	
Present	26 (35.6)
Absent	47 (64.4)

Values are presented as *n* (%).

**Table 4:** Antiresorptive treatment characteristics according to treatment indication.

Antiresorptive Treatment Characteristic	Oncologic (*n* = 73)	Osteoporosis (*n* = 46)
Antiresorptive agent, *n* (%)		
Zoledronic acid	42 (57.5)	0
Denosumab	31 (42.5)	0
Risedronate	0	6 (13.0)
Alendronate	0	25 (54.3)
Ibandronate	0	15 (32.6)
Route of administration, *n* (%)		
Intravenous	42 (57.5)	0
Subcutaneous	31 (42.5)	0
Oral	0	46 (100.0)
Dose, *n* (%)		
Zoledronic acid, 4 mg	42 (57.5)	0
Denosumab, 120 mg	31 (42.5)	0
Risedronate, 35 mg	0	6 (13.0)
Alendronate, 70 mg	0	25 (54.3)
Ibandronate, 150 mg	0	15 (32.6)
Administration frequency, *n* (%)		
Every 3 weeks	17 (23.3)	0
Every 4 weeks	56 (76.7)	0
Weekly	0	31 (67.4)
Monthly	0	15 (32.6)
Treatment duration (months), median (IQR)	11 (9–14)	42 (34–47)

Values are presented as *n* (%) or median (interquartile range [IQR]), as appropriate.

**Table 5:** Clinical characteristics and severity of MRONJ according to treatment indication.

Variable	Oncologic (*n* = 73)	Osteoporotic (*n* = 46)	*p*-Value
Anatomical location, *n* (%)			**0.037**
Mandible	49 (67.1)	22 (47.8)	
Maxilla	24 (32.9)	24 (52.2)	
AAOMS stage, *n* (%)			**0.001**
Stage I	17 (23.3)	23 (50.0)	
Stage II	32 (43.8)	19 (41.3)	
Stage III	24 (32.9)	4 (8.7)	
Advanced MRONJ (AAOMS II–III), *n* (%)	56 (76.7)	23 (50.0)	**0.003**
Extraction-related MRONJ, *n* (%)	48 (65.8)	32 (69.6)	0.666
Non-extraction-related MRONJ, *n* (%)	25 (34.2)	14 (30.4)	

Values are presented as *n* (%). Statistically significant *p*-values are shown in bold.

**Table 6:** Duration of antiresorptive treatment according to AAOMS stage.

AAOMS Stage	*n*	Duration of Antiresorptive Treatment (Months), Median (IQR)
I	40	26 (11–43)
II	51	15 (12–34)
III	28	11 (10–16)

Values are presented as median (interquartile range [IQR]).

**Table 7:** Univariable logistic regression analysis of factors associated with advanced MRONJ.

Variable	OR	95% CI	*p*-Value
Age, per 1-year increase	1.08	1.02–1.13	**0.006**
Vitamin D, per 1-unit increase (µg/L)	1.01	0.95–1.08	0.721
Treatment indication: osteoporotic vs. oncologic	0.30	0.14–0.67	**0.003**
Smoking, yes vs. no	1.05	0.45–2.46	0.915
Diabetes mellitus, yes vs. no	10.87	3.09–38.18	**<0.001**
Location: maxilla vs. mandible	0.47	0.22–1.02	0.056
Tooth extraction-related MRONJ, yes vs. no	0.47	0.20–1.13	0.093

Values are presented as odds ratios (ORs) with 95% confidence intervals (CIs). The reference categories were oncologic indication, no smoking, no diabetes mellitus, mandibular location, and non-extraction-related MRONJ. Statistically significant *p*-values are shown in bold.

**Table 8:** Multivariable logistic regression analysis of factors associated with advanced MRONJ.

Variable	OR	95% CI	*p*-Value
Age, per 1-year increase	1.16	1.06–1.27	**0.001**
Vitamin D, per 1-unit increase (µg/L)	1.04	0.96–1.13	0.376
Treatment indication: osteoporotic vs. oncologic	0.14	0.05–0.44	**<0.001**
Smoking, yes vs. no	0.98	0.32–2.98	0.969
Diabetes mellitus, yes vs. no	8.88	2.29–34.50	**0.002**
Location: maxilla vs. mandible	0.38	0.13–1.09	0.071
Tooth extraction-related MRONJ, yes vs. no	1.67	0.47–6.00	0.430

Values are presented as odds ratios (ORs) with 95% confidence intervals (CIs). The reference categories were oncologic indication, no smoking, no diabetes mellitus, mandibular location, and non-extraction-related MRONJ. Statistically significant *p*-values are shown in bold.

**Table 9:** Sensitivity analysis additionally adjusted for antiresorptive treatment duration.

Variable	OR	95% CI	*p*-Value
Age, per 1-year increase	1.16	1.06–1.27	**0.001**
Vitamin D, per 1-unit increase (µg/L)	1.04	0.96–1.13	0.369
Treatment indication: osteoporotic vs. oncologic	0.12	0.02–0.86	**0.035**
Smoking, yes vs. no	0.99	0.32–3.03	0.985
Diabetes mellitus, yes vs. no	8.89	2.29–34.53	**0.002**
Location: maxilla vs. mandible	0.38	0.13–1.08	0.069
Tooth extraction-related MRONJ, yes vs. no	1.67	0.47–5.99	0.431
Treatment duration, per 1-month increase	1.01	0.95–1.06	0.836

Values are presented as odds ratios (ORs) with 95% confidence intervals (CIs). The reference categories were oncologic indication, no smoking, no diabetes mellitus, mandibular location, and non-extraction-related MRONJ. Statistically significant *p*-values are shown in bold.

**Table 10:** Multivariable logistic regression analysis of factors associated with stage III MRONJ.

Variable	OR	95% CI	*p*-Value
Age, per 1-year increase	1.21	1.07–1.37	**0.002**
Vitamin D, per 1-unit increase (µg/L)	0.97	0.89–1.06	0.530
Treatment indication: osteoporotic vs. oncologic	0.08	0.02–0.32	**<0.001**
Smoking, yes vs. no	1.20	0.38–3.82	0.761
Diabetes mellitus, yes vs. no	1.35	0.49–3.72	0.566
Location: maxilla vs. mandible	0.40	0.14–1.16	0.091
Tooth extraction-related MRONJ, yes vs. no	3.92	1.01–15.20	**0.048**

Values are presented as odds ratios (ORs) with 95% confidence intervals (CIs). The reference categories were oncologic indication, no smoking, no diabetes mellitus, mandibular location, and non-extraction-related MRONJ. Statistically significant *p*-values are shown in bold.

## Data Availability

The data presented in this study are available from the corresponding author upon reasonable request.

## Abbreviations

The following abbreviations are used in this manuscript:

AAOMS	American Association of Oral and Maxillofacial Surgeons
AIC	Akaike information criterion
AUC	Area under the receiver operating characteristic curve
BIC	Bayesian information criterion
CI	Confidence interval
MRONJ	Medication-related osteonecrosis of the jaw
OR	Odds ratio
VIF	Variance inflation factors
